# Mistake is to Myth What Pretense is to Fiction: A Reply to Goodman

**DOI:** 10.1007/s11406-017-9812-5

**Published:** 2017-03-08

**Authors:** Björn Lundgren

**Affiliations:** Department of Philosophy and History, Division of Philosophy, KTH Royal Institute of Technology, Brinellvägen 32, 100 44 Stockholm, Sweden

**Keywords:** Creationism, Mythical abstracta, Fictional abstracta, Kripke, Ontology

## Abstract

In this reply I defend Kripke’s creationist thesis for mythical objects (*Reference and Existence*, 2013) against Jeffrey Goodman’s counter-argument to the thesis (“Creatures of fiction, objects of myth”, *Analysis, 74*(1), 35–40, 2014). I argue that Goodman has mistaken the basis for when mythical abstracta are created. Contrary to Goodman I show that, as well as how, Kripke’s theory consistently retains the analogy between creation of mythical objects and creation of fictional objects, while also explaining in what way they differ.

In a recent paper, Jeffrey Goodman presents a simple but seemingly efficient argument against a few philosophers’ theses on the creation of mythical objects (2014).^1^ One of these philosophers is Kripke; I will defend his creationist thesis against Goodman’s argument.^2^

I will explain the relevant details of Kripke’s specific creationism as this reply evolves, however – in general – a creationist is someone who believes in the existence of contingently created abstracta, e.g., a fictional creationist believes in the existence of the fictional abstracta such as Sherlock Holmes, while a mythical creationist believes in the existence of mythical abstracta such as unicorns (cf. p. 35).

Goodman’s basic argument rests on two theses:

- (1) If Vulcan is a created abstractum (like Holmes), then Vulcan is created by Le Verrier in every possible world where Le Verrier performs relevantly similar activities to those he actually performed.
- (2) There is a possible world where Le Verrier performs relevantly similar activities to those he actually performed and yet fails to create Vulcan (p. 37).

Goodman thinks that in some possible world, X, Le Verrier performs relevantly similar activities – as he did in the actual world – but in X “Le Verrier’s scientific hypothesis regarding an intra-Mercurial planet is true” (p. 38). Therefore, in X, (the actions of) Le Verrier would not have created a mythical planet, and thus (according to premise 1) the creation thesis must be false. As a comparison, if there is a flesh-and-blood person that matches with the description of Sherlock Holmes in the story, then this would not affect the creation of the abstracta Holmes (p. 37f).

I agree that in the former counter-factual situation the abstractum Vulcan is not created, but I deny that a creationist must accept premise 1. Premise 1 rests on the supposition that creation of myth is dependent only on authorial activities, not on facts about the mind-independent world (i.e. the world that is external to the author), which is just not true on Kripke’s theory. Firstly, on Kripke’s theory the creation of myth depends on *the author* (in Goodman’s words) being *mistaken* – a property which is normally dependent on the mind-independent world. When a five year old kid thinks that there is a ghost living under her bed and names it ‘Ghosty’ it is her mistake – and the mistaken usage of an empty name (‘Ghosty’) – that is the essential aspects of creation. Since *mistake* is the necessary qualifier it follows that premise 1 contains a non-sequitur since the creation of mythical abstracta in other possible worlds does not depend on *the author* performing relevantly similar activities to those she actually performed.^3^ Secondly, but related to the previous point, premise 1 is misleading because it makes it seem as if myth is necessarily created by authors – in the standard sense of an author – not by mistake. However, unlike fictional abstracta mythical abstracta are not necessarily created by an author and it is not necessary to identify any authorial intensions. Thus, while it is sensible to think of Le Verrier as an author, his authorial activities are not necessary for the actual creation of the myth. In fact, we need not even have an author involved in authorial activities for the actual criterion (which is something Goodman’s example and his text betrays). For example, Kripke considers cases in which the hearer (of a story) – or even the author (because she forgets) – mistakes a work of fiction for non-pretense (cf. Kripke 2013:30). In such cases – as in the case of Vulcan – the creation of the mythical abstractum is due to the mistake, not the authorial activities. In the previous example of the confused author the authorial activities are that of pretense and thus create fiction; the author does not create the myth because of any authorial activities (or as an author), rather the myth is created despite her authorial activities.^4^ Actual examples of this may include well-made mockumentaries, such as Peter Jackson’s *Forgotten Silver* (1996, New Zealand) in which Jackson is pretending to make a documentary in which he has rediscovered the filmmaker Colin McKenzie who was (according to the story) actually the first filmmaker to do tracking shots and close-ups. Unlike stories with the introductory “Once upon a time” Jackson is hiding his pretense, presenting it as a documentary. As such it is reasonable to think that some were tricked by Jackson, mistaking pretense for the real thing.^5^ If that is true, then a myth is created, but it is far-fetched to think of the people making these mistakes as being authors in the sense of Le Verrier. This implies that *the creation* of myth does not necessarily have anything to do with authorial activities at all, but is connected only to mistakes (cf. fn. 4, below). Thus, we should conclude that the specific example used by Goodman betrays the general nature of the creation of myth, which is dependent on mistake rather than authorial activities.

However, one may think that even though authorial activities are not necessary for *creation,* there may be special cases for which it is – such as for Vulcan. However, the buck does not stop here, because Goodman’s example and his text, is also misleading about his actual example, since not even *the creation* of the mythical object Vulcan is necessarily connected to Le Verrier authorial activities (cf. fn. 4, below). Consider the fact that in some possible world (albeit perhaps not amongst the closest ones) Le Verrier is writing the same theories as he actually did write, with the intensional difference that he merely pretends to be doing science or theorizing. In such a world, there exists a fictional entity which the name ‘Vulcan’ (in that world) refers to.^6^ However, imagine further that various people believe that Le Verrier is actually performing science and theorizing, just like people may have believed in *Forgotten Silver* (which is likely, presuming that this world is otherwise quite similar to ours). If so a mythical object named ‘Vulcan’ would be created, which arguably would be the same mythical object as in our world (however, in this possible world the name ‘Vulcan’ would be ambiguous). In such a case *the creators* (in the sense of being the cause of the creation) of the myth are the ones mistaking Le Verriers writing for being non-pretense, rather than Le Verrier himself. Clearly such a mistake is not authorial. What this example shows is that *the creation* of the mythical object Vulcan does not necessarily have anything to do with Le Verrier’s authorial activities at all, but rather with the mistake. A mistake which in the actual world happened to be his and part of his authorial activities, but in another possible world – in which his authorial intensions were otherwise – the mistake was not his. It needs to be noted that this example is not in direct conflict with premise 1, since Le Verrier’s authorial activities in the possible world in which he writes pretense are different from ours. Instead it is in conflict with premise 1 indirectly, since it shows that premise 1 is irrelevant, i.e. that Goodman’s argument is not a counter-example against Kripke’s creational theory, since creation of mythical abstracta are not related to authorial activities in the sense presumed.^7^ However, we could also imagine Le Verrier doing science, but not believing in his findings (for some reason). In such a situation Le Verrier would, arguably, be performing similar activities as he did, without creating the abstractum Vulcan – which would directly conflict with premise 1.

I will get back to the details of this in just a moment, for while Goodman does not re-count the specificity of Kripke’s theory (nor these more complex considerations) he does recognize the more general idea that premise 1 hangs on the thesis that created abstracta are created solely in virtue of the “author’s intentions and social/historical context” (p. 39).^8^ Goodman considers the idea that the creation of a mythical object also depends on the mind-independent world, such that if no relevant entity is provided the creation occurs, otherwise not (ibid). He responds:

This reply, however, forces a very unhappy decision on the mythical creationist. On the one hand, suppose she maintains that the analogy between the creative process for both fictional characters and mythical objects is extremely tight. If she then further claims that it is the very absence of an intra-Mercurial planet that is facilitating the creation of Vulcan in our world, then she should amend her fictional creationism by claiming that it is the very absence of a flesh and blood, supremely clever crime solver called Holmes who is fond of cocaine and pipe-smoking, etc. that is facilitating the creation of Holmes in our world. But no fictional creationist should say that. The world providing such an entity matters *not at all* as to whether Doyle’s activities succeed in creating Holmes (ibid).

There are several problems with Goodman’s reply. Firstly, the main gust of his argument is the idea that denying premise 1 solely for myth (but not for fiction) would imply a disanalogy between the creation of fictional entities^9^ and creation of mythical entities that would be unsatisfactory for the creationist. However, it is not clear why this would be the case. In fact, Goodman seems to be mistaken about the precise nature of how the creation of myth is analogous to the creation of fiction (according to the theories of Kripke). As previously noted Goodman has connected the creation of myth to authorial activities, in fact he seems to mistakenly believe that such a relation is necessary, thinking that mythical creationism is an expansion of fictional creationalism, in which the latter is “dependent on […] authorial activities” and the former – i.e. mythical abstracta “are likewise abstracta of our production” (p. 35). He furthermore establishes this link by nothing that “it is natural to think that Holmes came about *because* Doyle thought him up, and Vulcan came about *because* Le Verrier thought it up” (p. 36). But as my previous examples show it is not clear that this is the best way to interpret Kripke’s theory. As I have argued the fact that the mythical abstractum Vulcan came about *because* Le Verrier thought it up is only accidental (had his scientific writing been pretense, and mistaken for scientific writing, someone else would have been the creator of Vulcan). And this mistake – on Goodman’s part – is, arguably, what makes the argument of this so-called disanalogy seem sensible. However, it is rooted in a misconception of the difference between creation of myth and the creation of fiction and what necessarily differentiates one from the other, for they are not analogous in the authorial sense.

Secondly, the actual analogy between myth and fiction certainly is not perfectly similar, simply because myth and fiction are not the same. But just as myth is different from fiction, the creation of such abstracta differs. Thus, the analogy holds *only* in relation to these differences (as it should). As previously noted what Kripke claims is that mythical objects – such as Vulcan – are created in virtue of some mistake(s), and fictional objects are created in virtue of some pretense. Therefore, in accordance with these differences the analogy holds perfectly:

- A mythical object is created (partly) because of a mistake.
- A fictional object is created (partly) because of a pretense.

Given Kripke’s theory it is clear that when Goodman previously described a counter-factual situation in which Le Verrier was *not mistaken*, it is correct to say that in such a situation a mythical object was *not created*. However, Goodman seems to think that under such a theory the analogous situation is one in which there is a flesh-and-blood person matches the description of Doyle’s stories. This is not the case, for in such a situation it does not follow that Doyle is *not pretending*. Whether Doyle pretended is logically independent of whether a flesh-and-blood character exists.^10^ In fact, the example considered by Goodman is quite similar to Kripke’s criticism of descriptivism about naming, since for a descriptivist if there is an object that matches properly with the story then Sherlock Holmes exists (cf. Kripke 2013:26f).^11^ The analogous situation would instead be one in which Doyle is not pretending. Such a counter-factual situation, in which Sherlock Holmes is *not* created, was also previously presented by Kripke. He considers the possibility that he was wrong about Sherlock Holmes, i.e. that Doyle did not write stories but, e.g., newspaper articles: “If so, I am wrong, and I am under a mistaken impression that there are no such genuine propositions such as that Sherlock Holmes lived on Baker Street […] It may turn out for all that I know that I *have* made such a mistake” (Kripke 2013:42).

That is, under such a counter-factual situation, the fictional character Sherlock Holmes is *not created* and in such a possible world the name ‘Sherlock Holmes’ does not refer to the same object (nor the same kind of object) as the name ‘Sherlock Holmes’ does is in the actual world (given that we are correct in presuming that Sherlock Holmes is a fictional character, in the actual world).^12^

Returning to the question of the analogy between myth and fiction Kripke claims – as the title of this article indicates – that “most of what I say about pretense, though not perhaps all (you can check it out for yourselves), will apply *mutatis mutandis* with the term ‘mistake’ in place of ‘pretense’” (Kripke 2013: 31). This is the standard analogy between fiction and myth. Part of the problem with Goodman’s argument is that he presumes that the analogy must be stronger, so that premise 1 generally applies to both, but it is not clear if this presumption is compatible with a meaningful difference between myth and pretense, since for Goodman’s argument to work it would need to be presented against a theory in which mistake is replaced by another predicate that has similar authorial properties as pretense. It is questionable if such a creational theory would really even be about myths. Thus, it is questionable if any creational theory (at least any theory which adheres to the theory of direct references about proper names) is affected by Goodman’s argument; however, clearly Kripke’s theory is not.

To make it even more apparent that – contrary to Goodman – the analogy between myth and fiction still holds, consider Kripke’s previous example of the counter-factual situation in which Sherlock Holmes (the fictional character) does not exist: i.e. a possible world in which Doyle is not writing fiction, but a newspaper article about a flesh-and-blood detective named ‘Sherlock Holmes’. However, further imagine that no such flesh-and-blood detective exists. In this possible world, Doyle is not pretending, but he is mistaken. Therefore, in such a possible world a mythical abstractum named ‘Sherlock Holmes’ is created.

Thus, that the analogy holds does not mean that the situations of creation of fictional abstracta and mythical abstracta do not differ; they do. We can generalize the above examples to further illustrate how the theory hangs together. Imagine a text that includes the name ‘O’, which the text refers to as a physical object. If the text is pretense then ‘O’ (outside the text^13^) refers to a created abstractum in the form of a fictional object, if the text is neither pretense, nor mistake, then ‘O’ (outside, and inside, the text) refers to a physical object, and if the text is a mistake then ‘O’ (outside the text) refers to a created abstractum in the form of a mythical object.

This means that under normal circumstances the creation of myth is partly dependent on the mind-independent world, since whether we are mistaken about the non-mythical existence of Vulcan, unicorns, or Ghosty, is a questions dependent on what exists in the mind-independent world, and it is partly in virtue of the non-existence of such a non-mythical entity that the mythical objects come to exist. Fictional objects, on the other hand, do not – under normal circumstances – depend on the mind-independent world (at least not in the same sense), but on the question of whether the author actually pretended or not.

Thus, the relevant similarity is not that the process of mistake and the process of pretense are identical, but that both processes create an abstractum due to, e.g., the use of an empty name, and does so in a manner that is analogous in relation to itsdifferences (i.e. if the use of that empty name is due to a mistake, or pretense).^14^

As should be evident, Goodman has not compared a situation of non-pretense with a situation of non-mistake, but a situation of pretense with a situation of non-mistake. The seemingly disanalogous result that follow from Goodman’s comparing different forms of creationism is simply due to the use of disanalogous examples, and it does not affect Kripke’s theory in the least (in fact, the fictional example can be used in favor of Kripke’s account, by showing the downfall of descriptivism as applied to names in fiction).

The conclusion here is that premise 1 does not generally hold for creation of abstracta, since it standardly fails for myth. However, it is worth mentioning that there are plausibly some special cases in which premise 1 holds for myth, but in such cases premise 2 fails, so the argument does not go through anyway.^15^

## Footnotes

1 All references to Goodman are given henceforth with pagination only.

2 Goodman refers to Kripke (1973), the unpublished (transcript) of Kripke’s lecture Reference and Existence. In 2013 it was published and Kripke notes in the intro that he “did not intend to apply the notion [i.e. that the existence of fictional and mythical characters depends on the existence and non-existence of various fictional or mythological works], to ‘Vulcan’, ‘phlogiston’, or other vacuous theoretical names of a more recent vintage, which are ‘mythological’ objects only in a highly extended and perhaps even metaphorical sense of ‘mythological’ (Kripke 2013:x, the parenthesis is added by me as a paraphrase from Kripke, cf. Goodman 2013:37, fn. 7). I will avoid holding this clarification of Kripke’s against Goodman, partly because the clarification is an obvious addition to the original lecture, but mainly since Kripke continues to say that he is “not entirely sure of the difference in principle [---] So perhaps I should have extended the treatment to them as well” (Kripke 2013:x) and because the example of Vulcan is also discussed briefly by Kripke in the actual text (2013: 30f).

3 There is a slight complication. The simple case is this: in a possible world such that the five year old kid (presuming rigidity) performs the relevantly similar activities, and is not mistaken, she would not create a mythical abstractum called ‘Ghosty’, but rather she would name *something* ‘Ghosty’. What is slightly more complicated is that this *something* is not, according to Kripke, identical to what we would call Ghosty in our world (which is also why I use the word ‘something’ rather than ‘ghost’ for ghosts does not exist in any possible world, given that we are right in supposing that ghosts are actually only mythical abstracta and not a natural kind). Kripke gives a few arguments for this; the most important perhaps being that there is no way to qualify if a mythical abstractum is identical to some real thing. Even if we have descriptions of ghosts, or of Ghosty, any match with such description may be purely accidental – there is no way to know. It is for science to find out what actually qualifies as a natural kind, so just because something matches with the description of a mistake does not mean that it is identical with that mythical abstractum (given that it actually is a mythical abstractum) (cf. Kripke 2013: 41–53). Now, one may think that Vulcan would be a case contrary to this example. However, Le Verrier could have been right – in his existential claim – while being wrong about various descriptive qualities, so any likeness between Vulcan and objects named ‘Vulcan’ in worlds were Le Verrier performs similar activities as in our world and is right is thus purely accidental and insufficient for identity.

4 This should not be confused with the role that the authorial activities play in providing material for a mistake. However, as the thought example by Kripke shows, the actual mistake (i.e. the creational aspect) does not depend on the authorial activities as such, since it is, arguably, independent of the author’s authorial intension (which were that of pretense).

5 I say that is it reasonable since I want to avoid claiming it was actually the case, since there are myths about people believing in fiction. For example, people have mistakenly believed that Orson Welles fictional radioshow *War of the Worlds* was mistaken for the real thing (Chilton 2016).

6 This is not a refusal of rigidity, since it is important not to confuse the fact that in some possible world a combination the combination of symbols that make up the name ‘Saul Kripke’ does not necessarily refer to Saul Kripke in that world, since different languages and possible worlds have different symbol conventions. This is not to say that ‘Saul Kripke’ does not refer to Saul Kripke in every possible world.

7 Again, it should be noted (cf. fn. 4) that authorial activities often play a role in the creation of myths in so far as the authorial activities supply content of which we can be mistaken. But this is not the creational aspect. For example, Jackson’s authorial activities – i.e. that of making pretense (even though it, likely, was meant to be misleading) – were of course not what *created* the myth (if people actually mistook *Forgotten Silver* for a real documentary).

8 Goodman also quotes David Braun, in a footnote. In the quote Braun identifies mistakes as a necessary property for the creation of myths saying that “if story-tellers’ activities create fictional characters, then mistaken theorizers’ activities create abstract objects of a similar sort” (p. 36, fn. 5, Braun 2005: 615, emphasis added).

9 In this article I am using ‘entity’ and ‘object’ interchangeably.

10 Even if the existence of something which matches his descriptions would make it more likely that he did not make-up Sherlock Holmes.

11 This is perhaps not so surprising, since Goodman acknowledges that he “owe a huge debt to Phillips (2001) […] [who] aims to show that the direct reference theorist may not plausibly identify the referent of ‘Vulcan’ with a created abstractum – much of what [Phillips] says inspired the argument I present here against mythical creationism” (p. 37, fn. 6). According to Kripke, however, given that Sherlock Holmes *is* a fictional character “he” (in this article I ignore the fact that many philosopher’s – amongst which Kripke is probably one of them – would say that it is wrong to say he or she of a fictional character outside the story) could or would not he have been a flesh and blood person (cf. Kripke 2013:40ff). It should be noted that the implication above does not presume that Sherlock Holmes is a fictional character, neither does Kripke. Instead, whenever Kripke discusses cases of myth and fiction he keeps putting in caveats such as “remember here that we are supposing that there are no unicorns” (Kripke 2013:44) or “Given that this is so” (Kripke 2013:46), and it is given such presumptions that he discusses what could or would have been the case – and in virtue of such suppositions being true he can speak specifically about examples such as Sherlock Holmes. As such he would agree with Goodman that no flesh-and-blood entity can ever be identical with Sherlock Holmes (given that Sherlock Holmes *is* a fictional character), but the more general principle at work here is that no fictional character could or would [possibly] be a real flesh-and-blood person.

12 I would argue that – if we are true Kripkeans – what we should say about the name ‘Sherlock Holmes’ in that possible world and ‘Sherlock Holmes’ in the actual world, is that they are not the same names, since any name necessarily refers to the same objects in all possible worlds. Their similarity is merely a matter of symbolic identity; just as words in different languages can be constructed out of identical symbols while differing in meaning.

13 I have omitted the complications of what a name refers to within-the-story vs. outside-the-story (speaking only of the latter, since we have been speaking of abstractums which lies outside any text). Kripke’s theory is as follows: ‘Sherlock Holmes’ refers, in the actual world, to a fictional object (or character), while within-the-story ‘Sherlock Holmes’ refers to a flesh-and-blood person (a very clever detective) (see, e.g., Kripke 2013: “Lecture III”).

14 I am not saying that the creation of abstractums necessarily depends on naming, it may be the case that various abstractums may be created, e.g., as in the case of fiction when objects are implicitly spoken of within the pretense, without being named. Nothing hangs on this issues, it is only for the sake of simplifying the argumentation in this reply that I am using names.

15 For example, there are, arguably, cases in which mistake does not depend on anything else than the authorial activities. Such examples may include mistaken beliefs in impossibilities of an abstract nature, such as the belief that squares could be round or that 2 + 3 = 4. Now, whether such beliefs can be used in a creational manner without taking the mind-independent world into account depends on various ontological considerations. But this may serve as examples in which premise 1 holds for mythical creationism. However, since we are dealing with creation of impossibilia there are no possible world in which such beliefs are true. There is possibly an analogous case for fiction (i.e. on in which premise 1 does *not* hold for fiction). Let us paraphrase Kripke’s previous example – in which Doyle was writing newspaper articles – and consider a situation in which Doyle was writing newspaper articles, but due to heavy opium smoking (I apologies to any surviving relatives, or otherwise engaged people, that may take offence by my example) he was confused and thought that he was writing fiction. Presuming that author’s intentions are not everything (i.e. presuming that authors can fail) it would be intuitive to say that Doyle, in some such possible worlds, performs activities that are relevantly similar to those he *actually* performed, but fails to write fiction. I am here assuming that we all agree that in general we can create certain entities without intending to do so. Those who argue otherwise would of course need to counter the so-called ‘intentional fallacy’. In such a world, the created abstractum Sherlock Holmes does not exist. I’ve realized that intuitions go apart on this example (thanks to an anonymous reviewer for pointing that out), but the idea here is that Doyle could be performing relevantly similar activities in the sense that he tries to write fiction, he thinks he is pretending, but due to his confusion he is actually not. This may, on the other hand (if it is convincing) provide a problem for fictional creationism. Or, they can simply recognize that this is a special exception to the standard case, and that premise 1 is not even necessary for fiction (even if standardly true).
